# STC2 promotes anoikis resistance by modulating TGIF1 mRNA stability in colorectal cancer

**DOI:** 10.3389/fcell.2025.1695361

**Published:** 2026-01-29

**Authors:** Fan Hu, Qiuming He, Zheyu Ding, Jie Cheng, Jun Lin

**Affiliations:** 1 Department of Gastroenterology, Zhongnan Hospital of Wuhan University, Wuhan, Hubei, China; 2 Department of Gastroenterology, The Central Hospital of Wuhan, Tongji Medical College, Huazhong University of Science and Technology, Wuhan, Hubei, China; 3 Department of Gastrointestinal Surgery, Hubei Cancer Hospital, Tongji Medical College, Huazhong University of Science and Technology, Wuhan, Hubei, China

**Keywords:** anoikis, colorectal cancer, RNA stability, STC2, TGIF1

## Abstract

**Background:**

Stanniocalcin-2 (STC2), a glycosylated protein originally identified in the endocrine glands of fish, plays multiple biological roles in cancer. However, its functional significance and molecular mechanisms in colorectal cancer (CRC) remain unclear.

**Methods:**

Bioinformatic analyses and CRC tissue specimens were used to determine STC2 expression and its prognostic value. The biological effects of STC2 on CRC cells were assessed using flow cytometry and live/dead staining assays. The underlying mechanisms were further explored by RNA sequencing, RNA immunoprecipitation (RIP), and RNA stability assays.

**Results:**

STC2 was significantly upregulated in CRC tissues and cell lines, and its high expression was associated with poor prognosis in CRC patients. Functional experiments demonstrated that STC2 enhanced CRC cell resistance to anoikis by upregulating TGIF1 expression. Mechanistically, STC2 bound to TGIF1 mRNA and stabilized it by inhibiting its degradation.

**Conclusion:**

Our findings suggest that STC2 promotes anoikis resistance in CRC by regulating TGIF1 mRNA stability. STC2 may serve as a potential therapeutic target and prognostic biomarker for colorectal cancer.

## 1 Introduction

Colorectal cancer (CRC) ranks as the second most common cancer globally in terms of incidence and third in terms of mortality. According to the Global Cancer Statistics 2020, CRC was responsible for an estimated 1.93 million new cases and 0.93 million deaths worldwide (Sung et al., 2021; Kanth and Inadomi, 2021; Siegel et al., 2023). Statistics indicate a rapid increase in the incidence and mortality of CRC, particularly in medium-to-high Human Development Index (HDI) countries (Arnold et al., 2017). Owing to the lack of distinctive symptoms in the early stages, a significant number of CRC patients are not diagnosed until the disease has reached an advanced stage (Dekker et al., 2019). Therefore, early diagnosis is crucial for the survival and prognosis of patients with CRC. Currently, the exact mechanisms underlying the initiation and progression of CRC are not fully understood. Consequently, it is of great significance to further explore the molecular biological mechanisms of CRC and identify novel molecular markers for its diagnosis, prognosis, and possible therapeutic strategies.

With rapid advances in bioinformatics analysis and high-throughput RNA sequencing technologies, an increasing number of novel molecular biomarkers have been identified. In this study, we focused on the STC2 gene, identified through comprehensive bioinformatics analyses using data from The Cancer Genome Atlas (TCGA) and Gene Expression Omnibus (GEO) databases. Stanniocalcin (STC), a homodimeric glycoprotein hormone (Qie and Sang, 2022), which consists of stanniocalcin 1 (STC1) and stanniocalcin 2 (STC2) (Jiang et al., 2023; Ishibashi et al., 1998), is involved in calcium and phosphate secretion and implicated in cancer and angiogenesis (McCudden et al., 2002; Lal et al., 2001; Oxvig and Conover, 2023; Kahn et al., 2000; Wang et al., 2025). Several studies have indicated that STC2 expression is upregulated in various cancers such as renal cell carcinoma (Wang et al., 2018), breast cancer (Muñoz and Lampe-Huenul, 2025), liver cancer (Li et al., 2024), and cervical cancer (Wang et al., 2015). Previous research has revealed that STC2 is implicated in tumor development and progression and is associated with a range of biological functions, including apoptosis, inflammation, and oxidative stress responses (Liu et al., 2022; Yeung et al., 2012; Li et al., 2021; Yu et al., 2024), suggesting that STC2 may serve as a promising biomarker for disease severity and a potential therapeutic target for patients. However, the expression level of STC2 and its role in CRC have not yet been studied.

Anoikis, programmed cell death that occurs when normal cells lose contact with the extracellular matrix (ECM) or neighboring cells, plays an important role in tumor metastasis (Shaw et al., 2025; Mei et al., 2020; Zhang et al., 2025; Qu et al., 2025). As a form of apoptosis, anoikis can inhibit normal cells from acquiring malignant potential by preventing cell proliferation at inappropriate locations, whereas tumor cells can resist anoikis, thus surviving after detachment from their primary site and traveling through the lymphatic and circulatory systems until they colonize distant organs (Rennebeck et al., 2005; Strauss et al., 2010; Taddei et al., 2012; He et al., 2025). Several studies have indicated that anoikis is closely associated with the occurrence and development of cancer (Han et al., 2023; Ye et al., 2020; Jin et al., 2018; Buchheit et al., 2015). Therefore, understanding the mechanisms that regulate anoikis resistance in CRC cells could reveal novel strategies for the clinical treatment of CRC.

In this study, we identified that STC2 was upregulated in CRC cells and correlated with poor prognosis in patients with CRC. We found that TGIF1 is a target gene of STC2 using RNA sequencing. Moreover, we confirmed that STC2 regulates anoikis resistance in CRC cells. Mechanistically, STC2 regulates TGIF1 by binding to the mRNA of TGIF1 and repressing mRNA degradation. Our study revealed that STC2 could be a potential therapeutic target for CRC treatment and provides new insights into the mechanism of CRC occurrence and progression.

## 2 Materials and methods

### 2.1 Patients and samples

Three pairs of CRC tissues and their adjacent normal colon tissues were obtained from the Zhongnan Hospital of Wuhan University. None of the patients had received any preoperative treatment. Written informed consent was obtained from all the patients. The study was approved by the Ethics Committee of the Zhongnan Hospital of Wuhan University. All tissue samples were snap-frozen in liquid nitrogen immediately and then stored at −80 °C.

### 2.2 Cell culture and cell lines

Four human CRC cell lines (LoVo, HT29, HCT116, and SW480) and one human colon epithelial cell line (NCM460) were obtained from American Type Culture Collection (ATCC, USA). The cells were cultured in RPMI 1640 medium (Gibco, USA) supplemented with 10% FBS (Gibco, USA) and 1% penicillin–streptomycin under a humidified atmosphere of 5% CO2 at 37 °C. The HT-29 cell line, originating from an adenocarcinoma of the rectosigmoid part of the intestine, was authenticated using Short Tandem Repeat (STR) profiling to confirm its identity.

### 2.3 Plasmids, siRNA and lentivirus

All small interfering RNAs (siRNAs) were designed and generated by GenePharma (Shanghai, China). The sequences of siRNA duplexes are listed in Supplementary Table S2. Lipo2000 reagent (Invitrogen) was used for cell transfection according to the manufacturer’s protocol. The plasmids and lentiviruses were constructed by GenePharma (Shanghai, China). Cells were plated for functional assays or harvested for RNA or protein correlation analysis 24 or 48 h after transfection. To construct stably transfected cell lines, lentivirus and polybrene (final concentration of 5 μg/mL, Sigma Aldrich, Cat#107689) were added to 25% confluent cells. Fresh DMEM containing 10% FBS was added 16 h after infection, and the medium was replaced with medium containing the appropriate antibiotics 48 h after infection. After screening with puromycin (Sigma-Aldrich, USA), stably transfected cell lines were obtained for subsequent experiments.

### 2.4 Reverse transcription quantitative polymerase chain reaction (RT-qPCR)

Total RNA was extracted from CRC cell lines or tissues using TRIzol Reagent (Invitrogen, USA), according to the manufacturer’s protocol. The total RNA concentration of each sample was measured using a Nanodrop 2000 ultramicroscopy spectrophotometer (Thermo Scientific, USA), and 1 μg of RNA was reverse-transcribed into cDNA according to the instructions of the HiScript® Q RT SuperMix for qPCR (+gDNA wiper) kit (Vazyme, Nanjing, China). RT-qPCR experiments were performed on a Bio-Rad IQ5 real-time PCR instrument (Bio-Rad, USA) and SYBR Green PCR Master Mix (Vazyme, Nanjing, China) in a 20 μL reaction system. The 2^−ΔΔCT^ method was used to calculate relative expression. Primer sequences used in this study are listed in Supplementary Table S3.

### 2.5 Western blot assay

Cells were lysed on ice in RIPA buffer containing protease inhibitor (Thermo Scientific, USA). Protein levels were quantified using bicinchoninic acid (BCA) assay after extraction. Next, the proteins were separated by sodium dodecyl sulfate-polyacrylamide gel electrophoresis (SDS-PAGE) and transferred to polyvinylidene fluoride (PVDF) membranes (Millipore, USA). After being blocked with 5% nonfat milk for 2 h in TBST, the membranes were incubated with primary antibodies overnight at 4 °C and then incubated with diluted HRP-conjugated secondary antibodies for 1 h at room temperature. Protein bands were detected using the Bio-Rad ChemiDoc XRS System, and band intensity was measured using Bio-Rad Image Lab software. The antibodies used in this study are listed in Supplementary Table S4.

### 2.6 Immunohistochemistry

CRC tissues were fixed in 10% neutral-buffered formalin for 30 min and subsequently embedded in paraffin. Serial sections (4 μm thick) were prepared using a microtome and mounted onto glass slides. Immunohistochemical (IHC) staining was performed following the manufacturer’s protocol. After staining, slides were scanned and analyzed using an automated digital pathology system (Aperio VERSA 8, Germany).

### 2.7 Colony formation

The proliferative capacity of STC2-KD and STC2-OE CRC cells was evaluated using colony formation and CCK-8 assays. For colony formation, cells were seeded in 6-well plates at a density of 1,000 cells/well and incubated for 2 weeks under standard culture conditions. Colonies were fixed with 4% paraformaldehyde for 30 min, stained with 0.5% crystal violet for 15 min, and rinsed gently with PBS. The colonies were imaged and quantified under a light microscope.

### 2.8 Scratch wound-healing assay

Cell migration ability was assessed using a wound-healing assay. STC2-KD and STC2-OE CRC cells were seeded into 6-well plates and cultured until reaching approximately 90% confluence. A straight wound was created across the monolayer using a sterile 10 μL pipette tip. Detached cells were removed by washing twice with PBS, followed by incubation in serum-free medium at 37 °C. Images of the wound area were captured at 0 h and 24 h, and migration rates were quantified using ImageJ software (NIH, USA).

### 2.9 Transwell migration and invasion assay

To evaluate the migration and invasion ability of STC2-KD and STC2-OE CRC cells, we performed Transwell migration and invasion assays using Transwell chambers (8 μm pore size, Corning, USA). The upper chambers of the transwell precoated with Matrigel (Corning, USA) were used for the invasion assay, while the migration assay was performed without Matrigel. CRC cells were seeded in the upper chambers and incubated in serum-free DMEM, whereas medium containing 10% FBS was placed in the lower chambers. After incubation for 48 h, the cells and Matrigel above the membrane in the upper chamber were removed, and the migrated and invaded cells on the lower surface of the membrane were fixed in 4% paraformaldehyde and stained with 0.5% crystal violet. Finally, five random fields were selected to count the cells and were photographed under a microscope (magnification, ×200).

### 2.10 RNA sequencing

Total RNA was extracted from the Control and STC2-KD groups of LoVo cells using TRIzol reagent (Invitrogen, USA), following the manufacturer’s instructions. Quality control, library construction, RNA sequencing, and bioinformatics analysis were performed at the Beijing Genomics Institute (BGI, Shenzhen, China) using the DNBSEQ platform. Briefly, raw sequencing reads were filtered to remove low-quality reads, adaptor sequences, and those containing poly-N. Clean reads were aligned to the human reference genome (GRCh38) using the HISAT2. Gene expression levels were quantified and normalized using the FPKM (Fragments Per Kilobase of transcript per million mapped reads) method. Differentially expressed genes (DEGs) between the two groups were identified using DESeq2, with a threshold of |log2 fold change| >1 and an adjusted p-value (FDR) <0.05.

Gene Ontology (GO) and Kyoto Encyclopedia of Genes and Genomes (KEGG) pathway enrichment analyses of the identified DEGs were performed using the Dr. Tom network platform provided by BGI (http://report.bgi.com).

### 2.11 Anoikis assay

The cells were plated in low-binding 6-well plates (Corning, USA) at a density of 150 cells/well. After 5 days, the cells were collected and a Live/Dead Cell Double Staining Kit (Abbkine, Wuhan, China) was used to determine cell viability. Under fluorescence microscopy, we observed that live cells were stained green, whereas dead cells were stained red.

### 2.12 Flow cytometry

The cells were plated in low-binding 6-well plates and cultivated for 5 days. The cells were processed using the Annexin V-FITC/PI double stain apoptosis detection kit, according to the manufacturer’s instructions (#4101–2; BestBio). Fluorescence intensity was detected using a flow cytometer (CytoFlex S, Beckman Coulter, Wuhan, China), and the results were analyzed using FlowJo software. Apoptotic rate was calculated as the percentage of early and late apoptotic cells.

### 2.13 RNA stability assay

To evaluate TGIF1 mRNA stability, Control and STC2-KD cells were treated with 5 μg/ml actinomycin D for 0, 3, 6, 9, and 12 h to extract RNA for RT-qPCR.

### 2.14 RNA binding protein immunoprecipitation (RIP)

To explore the correlation between STC2 and TGIF1, we performed a RIP assay using an RIP kit (Millibo, Massachusetts, USA). Briefly, CRC cells were collected and lysed using RIP lysis buffer. Then the anti-STC2 and anti-IgG antibodies were then added and cultured for 30 min, and total RNA (10 μL, input control) was used as a control. Next, the incubated samples were washed six times with the RIP wash buffer. Finally, RNA was extracted using the TRIzol method, reverse transcribed to cDNA, and measured by RT-qPCR.

### 2.15 *In vivo* tumor xenograft

Animal experiments were approved by the Wuhan University Ethics Committee. Six-week-old female BALB/c-nu mice were obtained from GemPharmatech (Jiangsu, China). All mice were raised in the standard animal facility room and randomly divided into two groups (Control, STC2-KD). To establish a subcutaneous xenograft model, 5.0 × 10^6^ cells were resuspended in 200 μL of serum-free DMEM and Matrigel (Corning, USA) (1:1) and subcutaneously injected into the right armpit of each mouse. After 35 days, the experimental mice were euthanized, and the subcutaneous tumors were resected, photographed, weighed, and subjected to statistical analyses.

### 2.16 Statistical analysis

All experiments were performed independently at least three times. Data are presented as the mean ± standard deviation (SD). Statistical analyses were conducted using GraphPad Prism (version 9.0; GraphPad Software, USA) and SPSS Statistics (version 22.0; IBM Corp., USA). Comparisons between two groups were assessed using a two-tailed Student’s t-test or chi-square test, as appropriate. Survival curves were generated using the Kaplan-Meier method and compared using the log-rank test. Univariate and multivariate Cox proportional hazards regression analyses were performed to identify independent prognostic factors. Differentially expressed genes (DEGs) were identified using the DESeq2 algorithm, which was applied to RNA sequencing data. Data normalization was performed using the Fragments Per Kilobase of transcript per Million mapped reads (FPKM) method. Statistical significance was assessed with a threshold of |log2 fold change| >1 and an adjusted p-value (FDR) < 0.05. A p-value <0.05 was considered statistically significant. (*p < 0.05, **p < 0.01, ***p < 0.001).

## 3 Results

### 3.1 STC2 is highly expressed in CRC tissues and predicts poor prognosis

**FIGURE 1:**
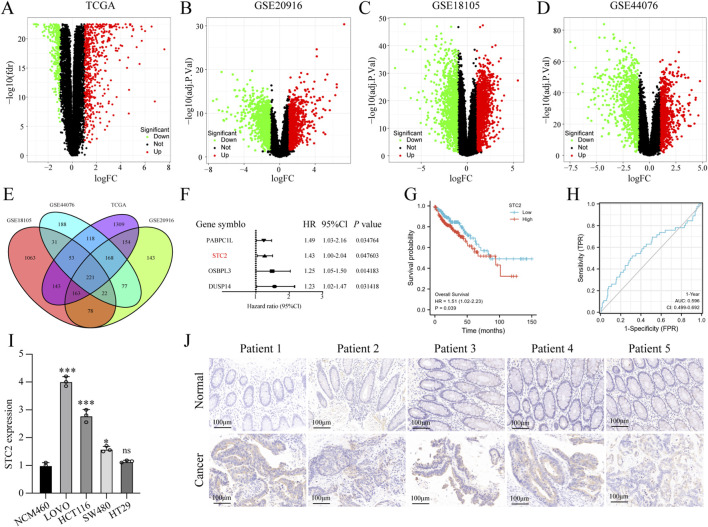
Upregulation of STC2 is associated with the progression of CRC. **(A)** Volcano plot showing differential expression genes (DEGs) from TCGA cohort. **(B–D)** Volcano plot showing DEGs from GSE cohort. **(E)** Venn diagram showing overlap between DEGs identified from TCGA database and GSE database. **(F)** The forest plot of four prognosis-related DEGs. **(G)** Kaplan-Meier survival curve for overall survival of CRC patients with low and high expression of STC2. **(H)** 1-year-prognostic ROC curve analysis to evaluate the prognostic value of SCT2 expression in CRC. **(I)** STC2 expression in normal colorectal epithelial cells and CRC cells lines. **(J)** Immunohistochemical (IHC) staining of STC2 expression in CRC tissues and peritumoral tissue. *p < 0.05; **p < 0.01; ***p < 0.001; ns, no significance.

To explore the pathogenesis of CRC and discover new ideas for the diagnosis and treatment of CRC, we used four datasets to identify differentially expressed genes (DEGs). Volcano plots showed that the DEGs were retrieved from TCGA, GSE20916, GSE18105, and GSE44076 (Figures 1A–D). By analyzing the intersection of the four datasets, we identified 221 upregulated DEGs (Figure 1E). We found four survival-related genes following the analysis of the relationship between upregulated gene expression and survival (Figure 1F). Among them, STC2 has been reported to be overexpressed in gastric cancer (Yokobori et al., 2010), breast cancer (Raulic et al., 2008), and hepatocellular carcinoma (Long et al., 2022), and its expression is positively correlated with cancer progression. However, the role of STC2 in CRC remains to be elucidated. Therefore, we chose STC2 as a target gene for further research and attempted to discover its role in CRC development. First, we found that the expression levels of STC2 were higher in CRC tissues than in normal tissues based on data from TCGA database, especially in tumors with higher stages (Supplementary Figure S1A,B). Moreover, STC2 expression was associated with T stage and N stage, but not with M stage, age, or sex (Supplementary Figure S1B-F). Univariate Cox regression analysis showed that STC2 expression was significantly associated with prognosis in TCGA (Supplementary Table S1). Kaplan-Meier curve analysis indicated that high STC2 expression was associated with poor clinical outcomes in CRC patients (Figure 1G). Logistic regression analysis was performed to assess the effectiveness of STC2 in the diagnosis of CRC. In the ROC curve of STC2, the mean area under the ROC curve (AUC) (0·596 ± 0·050) (Figure 1H) revealed that STC2 had good diagnostic value for CRC. Similarly, we confirmed that STC2 was significantly upregulated in the CRC cell lines (Figure 1I). In addition, immunohistochemistry (IHC) revealed that STC2 was highly expressed in CRC tissues compared to paired adjacent normal tissues (Figure 1J). Collectively, these results demonstrated that STC2 is highly expressed in CRC and can serve as a prognostic marker associated with poor outcomes.

### 3.2 STC2 promotes proliferation, migration, invasion in CRC cell lines

**FIGURE 2:**
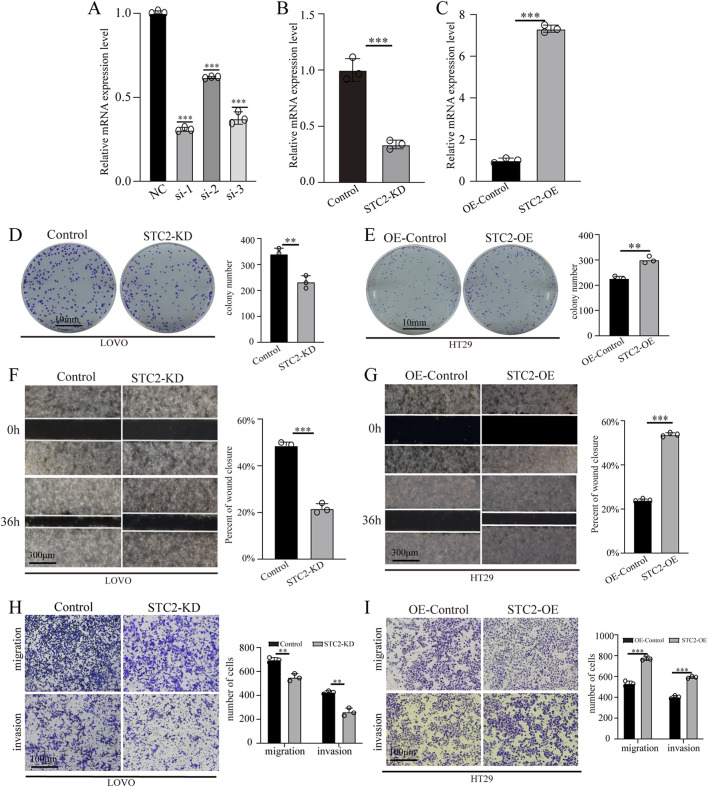
STC2 promotes proliferation, migration, invasion and cell cycle progression in CRC cell lines. **(A)** The knock-down efficiency of three si‐STC2 sequences was detected by RT-qPCR. **(B)** The efficiency of knockdown of STC2 in LoVo by the lentiviral vector. **(C)** The efficiency of overexpression of STC2 in HT29 by the lentiviral vector. **(D,E)** The clonogenic assay of CRC cell lines after STC2 knockdown **(D)** or overexpression **(E)**. **(F,G)** Wound healing experiment of CRC cell lines treated with STC2-KD **(F)** or STC2-OE **(G)**. **(H,I)** Migration and invasion assays of CRC cell lines after STC2 knockdown **(H)** or overexpression **(I)**. *p < 0.05; **p < 0.01; ***p < 0.001; ns, no significance.

RT-qPCR analysis revealed the highest expression level of STC2 in LoVo colon cancer cells and the lowest expression levels of STC2 in HT29 colon cancer cells. To further explore the role of STC2 in tumor progression, we selected these 2 cell lines for knockdown and overexpression experiments. First, three siRNA sequences were designed for STC2 knockdown, and si1 exhibited a better knockdown efficiency. (Figure 2A). Therefore, we chose si1 for subsequent experiments. STC2 stable knockdown and overexpression cell lines were constructed using a lentivirus vector, designated as STC2-KD and STC2-OE, respectively, in LoVo and HT29 cells (Figures 2B,C). The colony formation assay showed that STC2-KD inhibited CRC cell proliferation (Figure 2D), whereas STC2-OE had the opposite effect (Figure 2E). Transwell migration, invasion, and wound healing assays showed that the migration and invasion of CRC cells were inhibited by STC2-KD (Figures 2F,H). Conversely, STC2-OE induced migration and invasion of CRC cells (Figures 2G,I). Collectively, these results confirmed that STC2 promotes CRC cell proliferation, migration, and invasion.

### 3.3 STC2 overexpression inhibits anoikis in CRC cell lines

**FIGURE 3:**
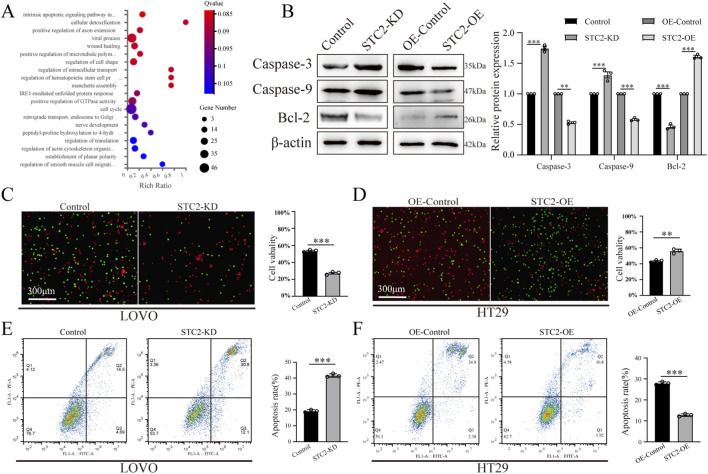
STC2 overexpression inhibits anoikis in CRC cell lines. **(A)** Bubble chart shows the KEGG pathway enrichment analysis of DEGs after STC2 knockdown. **(B)** WB analysis of Caspase-3, Caspase-9, and Bcl-2 in LoVo-NC and STC2-KD cell lines and in HT29-NC and STC2-OE cell lines. **(C)** Live and dead staining of LoVo-NC and STC2-KD cell lines after culture on 3D ultralow attachment plates for 5 days **(D)** Live and dead staining of HT29-NC and STC2-OE cell lines after culture on 3D ultralow attachment plates for 5 days **(E)** Flow cytometric analysis showing apoptosis rate of LoVo-NC and STC2-KD cell lines after culture on 3D ultralow attachment plates for 5 days **(F)** Flow cytometric analysis showing apoptosis rate of HT29-NC and STC2-OE cell lines after culture on 3D ultralow attachment plates for 5 days *p < 0.05; **p < 0.01; ***p < 0.001; ns, no significance.

To investigate the biological role of SCT2 in CRC, we performed RNA sequencing on STC2-NC and STC2-KD cells. The DEGs were then used to conduct Gene Ontology (GO) enrichment analysis of the biological processes, and the DEGs were primarily enriched in pathways related to cell survival and cell death (Figure 3A). To the best of our knowledge, tumor metastasis requires tumor cells to detach from primary sites to resist anoikis, a type of intrinsic apoptosis initiated by detachment from the ECM. Resistance to anoikis is considered an important marker of tumor metastasis and is essential for tumor progression (Haun et al., 2018; Wang J. et al., 2022). Thus, we explored whether STC2 regulates anoikis in CRC cells. First, we simulated the *in vivo* microenvironment of CRC cell detachment from the primary site. The cells were cultured on 3D ultralow attachment plates for 5 days. We then collected the cells and measured the expression of anoikis markers by Western blotting. The results indicated that STC2-KD markedly upregulated caspase-3 and caspase-9 expression and downregulated Bcl-2 expression in matrix-detached CRC cells, whereas STC2-OE had the opposite effect (Figure 3B). Moreover, cell viability measurements revealed that STC2 overexpression significantly enhanced the survival of matrix-detached CRC cells, whereas STC2-KD did the opposite (Figure 3C,D). Flow cytometry analysis also demonstrated that STC2-KD promoted CRC cell anoikis, whereas STC2-OE inhibited cell anoikis. (Figure 3E,F). Collectively, our results indicate that STC2 can significantly boost anoikis resistance in CRC cells.

### 3.4 STC2 upregulates the expression of TGIF1 in CRC cells

**FIGURE 4:**
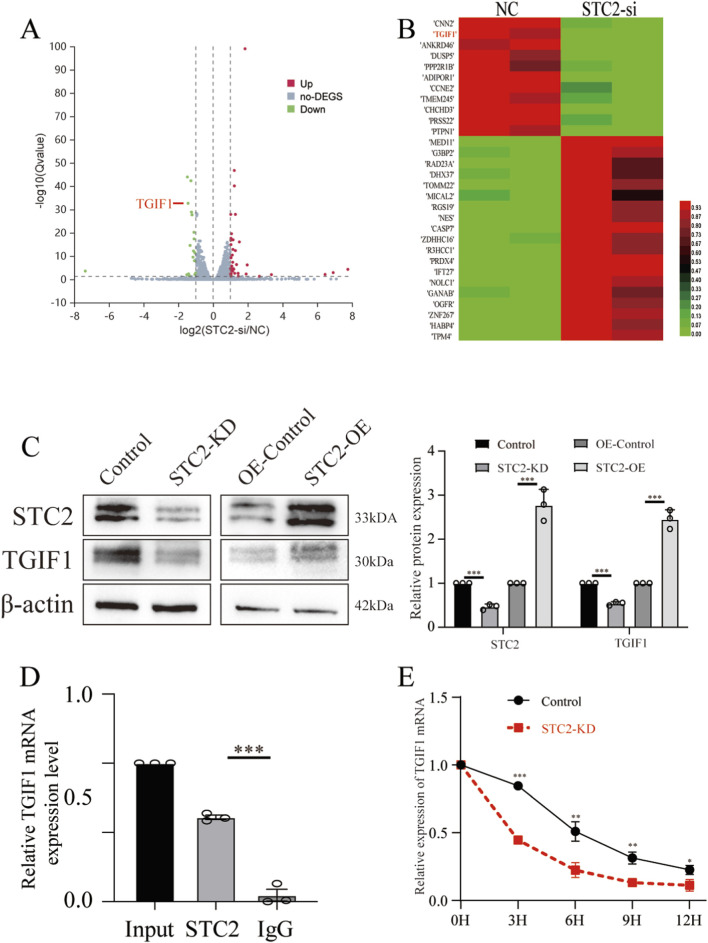
STC2 upregulates the expression of TGIF1 in CRC cells. **(A)** Differentially expressed genes in STC2-NC and STC2-KD groups. **(B)** Heatmap presenting significantly differentially expressed transcripts between LoVo-NC and STC2-KD cell lines. **(C)** WB analysis of STC2 and TGIF1 in LoVo-NC and STC2-KD cell lines and HT29-NC and STC2-OE cell lines. **(D)** RIP analysis conducted using an anti-STC2 antibody to validate the interaction between STC2 and TGIF1 mRNA. **(E)** TGIF1 mRNA decay line chart of LoVo-NC and STC2-KD cell lines after treated with actinomycin **(D)** *p < 0.05; **p < 0.01; ***p < 0.001; ns, no significance.

To explore the molecular mechanisms of STC2-mediated anoikis resistance, we re-analyzed our RNA sequencing in STC2-deficient and control LoVo cells to identify downstream target genes of STC2.A total of 33 DEGs, including 23 upregulated and 10 downregulated genes (Figure 4A, Supplementary data.1). Among them, TGIF1, one of the dysregulated genes, was significantly changed (Figure 4B). To further validate the relationship between STC2 and TGIF1 in CRC at the clinical level, we analyzed the correlation of their expression in two independent CRC cohorts. In the TCGA-CRC dataset, STC2 expression was significantly positively correlated with TGIF1 expression (Pearson’s r = 0.146, *p* < 0.001) (Supplementary Figure S2A). Similarly, in the GEO dataset GSE17538, the correlation was statistically significant (Pearson r = 0.295, *p* < 0.001) (Supplementary Figure S2B). These results suggested a consistent co-expression pattern of STC2 and TGIF1 across different clinical datasets, supporting the existence of a potential regulatory relationship between these two genes in CRC. A previous study reported that TGIF1 is associated with CRC carcinogenesis and progression (Shah et al., 2019). Thus, we selected TGIF1 for further investigation. Consistent with the RNA sequencing results, Western blot results demonstrated that STC2-KD caused notably decreased expression of TGIF1 in LoVo cells. Conversely, STC2-OE significantly increased the expression of TGIF1 in HT29 cells (Figure 4C). Immunofluorescence staining showed that STC2 was predominantly localized in the cytoplasm, with minimal nuclear signals (Supplementary Figure S2C). To examine whether STC2 can bind to TGIF1 mRNA, we performed RIP experiments using anti-STC2 in LoVo cell lysates. The results demonstrated that TGIF1 mRNA was specifically enriched using an anti-STC2 antibody, indicating that STC2 directly binds to TGIF1 mRNA (Figure 4D). In addition, CRC cells were exposed to actinomycin D (AD) (5 μg/mL) at the indicated time points and analyzed using RT-qPCR. The half-life of TGIF1 mRNA is reduced in STC2-KD cells. The results indicated that STC2-KD significantly affected TGIF1 mRNA decay in actinomycin D (Figure 4E). Taken together, these results suggest that STC2 promotes TGIF1 expression by regulating mRNA stability.

### 3.5 STC2 promotes anoikis resistance in CRC cells via TGIF1

**FIGURE 5:**
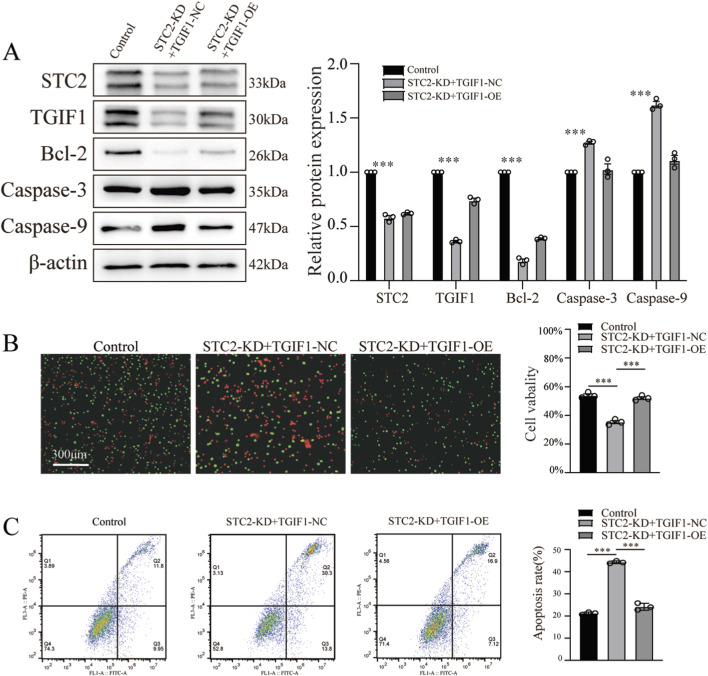
STC2 promotes anoikis resistance in CRC cells via TGIF1. **(A)** Western blot analysis of cleaved caspase-3, cleaved caspase-9, and Bcl-2 expression in matrix-detached LoVo cells. TGIF1 overexpression reversed the apoptosis-related protein changes induced by STC2 knockdown. **(B)** Cell viability of matrix-detached LoVo cells was assessed by Live and dead staining assay. TGIF1 overexpression significantly increased cell survival in STC2-KD cells. **(C)** Flow cytometry analysis of apoptosis in matrix-detached LoVo cells. TGIF1 overexpression reduced the proportion of apoptotic cells induced by STC2 knockdown.

To confirm whether TGIF1 mediates STC2-induced anoikis resistance in CRC cells, we performed a rescue experiment by overexpressing TGIF1 in STC2-knockdown LoVo cells. Compared to the STC2-KD group, TGIF1 overexpression reduced the expression of cleaved caspase-3 and cleaved caspase-9 and restored Bcl-2 expression under anchorage-independent conditions, indicating a reversal of pro-apoptotic signaling (Figure 5A). Moreover, cell viability assays demonstrated that TGIF1 overexpression significantly improved the survival of matrix-detached CRC cells in an STC2-KD background (Figure 5B). Consistently, flow cytometry analysis revealed that TGIF1 overexpression markedly reduced the apoptosis rate in STC2-deficient cells (Figure 5C). We also examined the expression of key anoikis-related proteins in CRC cells under TGIF1 modulation. LoVo cells were transfected with TGIF1-specific siRNA plasmids and cultured in matrix-detached conditions. WB analysis showed that TGIF1 knockdown resulted in significant upregulation of caspase-3 and caspase-9 and downregulation of Bcl-2, indicating increased activation of apoptosis (Supplementary Figure S2D). Collectively, these results indicated that TGIF1 functions as a critical downstream effector of STC2 and is capable of partially rescuing the anoikis-sensitive phenotype induced by STC2 knockdown in CRC cells.

### 3.6 STC2 promotes CRC tumorigenesis *in vivo*

**FIGURE 6:**
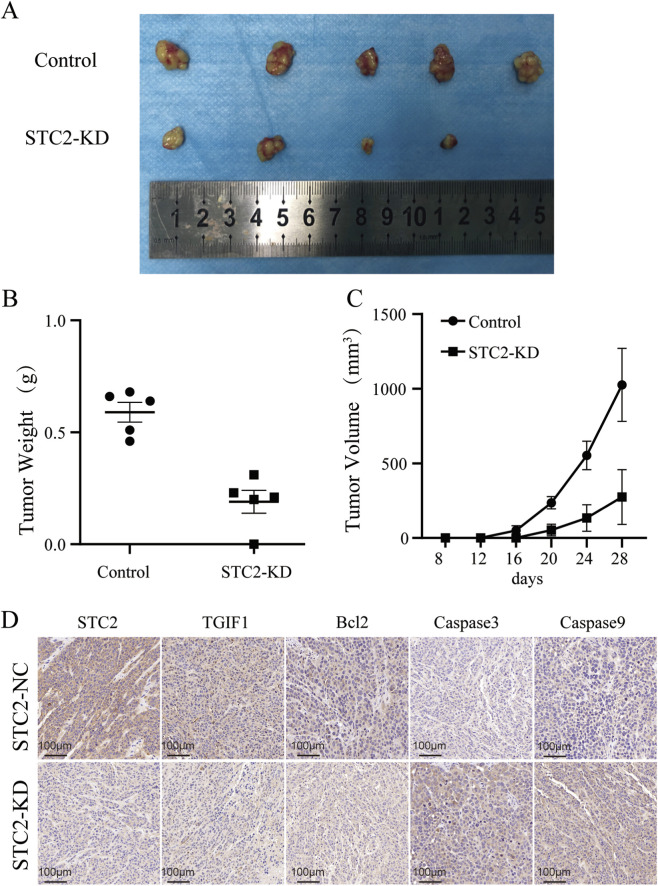
STC2 promotes CRC cell survival and metastasis *in vivo*. **(A)** The morphological characteristics of subcutaneous tumor xenografts in LoVo-NC and STC2-KD groups. **(B)** Subcutaneous tumor weight in LoVo-NC and STC2-KD groups. **(C)** Subcutaneous tumor volume in LoVo-NC and STC2-KD groups. **(D)** Immunohistochemistry of STC2, TGIF1, Caspase-3, Caspase-9, and Bcl-2 in tumor xenografts.

To further assess the effect of STC2 on CRC tumor growth *in vivo*, we established a nude mouse xenograft model. LoVo-KD-STC2 and the corresponding control cells were injected subcutaneously into nude mice. After 30 days, the mice were euthanized by cervical dislocation after anesthesia with pentobarbital sodium. Tumor tissues were collected for further analysis. As expected, the tumors in the STC2-KD group were significantly smaller and lighter (Figures 6A–C). IHC was performed to detect the expression of TGIF1 and anoikis-related factors in the xenograft tumors (Figure 6D). IHC staining confirmed that STC2 could regulate the expression of TGIF1 and anoikis *in vivo*. Thus, we conclude that STC2 promotes CRC growth *in vivo*.

## 4 Discussion

STC2 was initially identified as a glycosylated protein in the endocrine glands of fish and is primarily involved in calcium and phosphate transport (Ishibashi et al., 1998; Chang et al., 1995; Olsen et al., 1996; Liu et al., 2025). Recent studies have highlighted STC2’s involvement in the progression of various cancers, including gastric cancer (Fang et al., 2014), breast cancer (Esseghir et al., 2007), nasopharyngeal carcinoma (Li et al., 2022) and esophageal cancer (Kita et al., 2011), although the underlying mechanisms remain unclear. In our study, we demonstrated that STC2 is highly expressed in colorectal cancer (CRC) and its elevated expression is closely associated with metastasis and poor prognosis in CRC patients. Furthermore, we found that STC2 promoted CRC cell resistance to anoikis by stabilizing TGIF1 mRNA. Although STC2 is classically characterized as a secreted glycoprotein, increasing evidence demonstrates its intracellular activity. Consistent with prior findings that STC2 interacts with cytoplasmic proteins such as PRMT5 (Jiang et al., 2023), our immunofluorescence and RIP assays confirm cytoplasmic localization and RNA-binding potential. Our findings suggest that STC2 could serve as a potential therapeutic target for CRC treatment.

Recent evidence suggests that STC2 is involved not only in anoikis resistance but also in metabolic adaptation and stress-response pathways (Qie and Sang, 2022; Jiang et al., 2023; Lei et al., 2024), implicating potential interactions with the broader metabolic–immune landscape of CRC. Tumor metabolism and immune infiltration jointly shape the tumor microenvironment and influence metastatic competence, and future studies should explore how STC2 integrates into these networks. Moreover, although STC2 shows prognostic value, single-gene biomarkers generally lack the robustness of combined signatures. Integrated multi-gene immune–metabolism models typically outperform single markers (Xiao et al., 2022; Xiao et al., 2021). Therefore, STC2 may be most effective when incorporated into multi-parameter prognostic frameworks rather than used alone.

Anoikis is a specialized form of programmed cell death triggered by the detachment or improper adhesion of cells to the extracellular matrix. It plays a critical role in organismal development, tissue homeostasis, disease onset, and tumor metastasis (Sun et al., 2023; Kim et al., 2012). Anoikis prevents abnormal proliferation and distant metastasis of detached tumor cells (Zhang J. et al., 2023). Resistance to anoikis is often a hallmark of tumor invasion, metastasis, treatment resistance, and recurrence (Yu et al., 2022; Tlili et al., 2025). Recent studies have confirmed the involvement of anoikis in the initiation and progression of various cancers, including gastric cancer (Ye et al., 2020), endometrial cancer (Chen et al., 2018), and breast cancer (Alard et al., 2023). However, the precise mechanism through which STC2 regulates anoikis in CRC remains unclear. This study is the first to show that STC2 regulates anoikis in CRC cells by modulating TGIF1 mRNA stability and upregulating its expression. This study broadens our understanding of STC2’s role in cancer progression and tumorigenesis.

TGIF1 is a member of the three-amino acid loop extension (TALE) homeodomain protein family and is involved in various important cellular processes, such as proliferation, differentiation, and apoptosis (Zhang et al., 2020; Wang H. et al., 2022). Numerous studies have shown that TGIF1 functions through specific interactions with Smad proteins and other cofactors, influencing TGF-β signaling pathways (Guca et al., 2018). Hamid et al. reported that TGIF1 plays a significant role in both normal and malignant hematopoiesis, with TGIF1 knockdown inhibiting the proliferation and differentiation of leukemia cell lines (Ham et al., 2009). Xiang et al. demonstrated that TGIF1 contributes to the malignant progression of non-small cell lung cancer (NSCLC), where TGIF1 overexpression promotes cancer cell growth and migration (Xiang et al., 2015). However, unlike these studies, we identified TGIF1 as a key mediator of STC2-induced anoikis resistance, potentially through the regulation of apoptosis-related proteins by TGIF1. This insight provides a novel perspective on the mechanisms underlying anoikis disruption in CRC.

Gene expression regulation occurs at two fundamental levels: transcriptional and post-transcriptional (Montgomery and Dermitzakis, 2011; Zhang Q. et al., 2023; Bentley, 2014). Modulation of mRNA stability represents a crucial post-transcriptional event that significantly affects the mRNA pool available for translation (Keene and Tenenbaum, 2002). Our study revealed the role of oncogene mRNA stability regulation in cancer metastasis. Specifically, STC2 modulates the stability of TGIF1 mRNA by binding to its mRNA. Knockdown of STC2 reduced TGIF1 mRNA stability, indicating that STC2 helps stabilize TGIF1 mRNA by preventing its degradation. These findings expand our understanding of cancer gene regulation and provide new insights into the underlying molecular mechanisms.

However, our study had some limitations. While our RIP and mRNA decay assays support a stabilizing effect of STC2 on TGIF1 transcripts, future studies incorporating TGIF1 3′UTR luciferase reporter assays and mutational mapping will be essential to precisely define the specific cis-regulatory elements involved. Although we demonstrated that STC2 inhibits anoikis to promote CRC progression, STC2 may also influence CRC progression through other biological pathways, which warrants further investigation. Additionally, Targeting STC2 could provide a promising therapeutic strategy for CRC treatment. Potential approaches include monoclonal antibodies that specifically inhibit STC2 function or small molecule inhibitors designed to block its interaction with mRNA. While these strategies may offer substantial therapeutic benefits, potential side effects could include immune-related adverse events from monoclonal antibodies, or off-target effects from small molecules that may interfere with other RNA-binding proteins, thus necessitating further preclinical and clinical evaluation.

In conclusion, this study demonstrated that STC2 is upregulated in colorectal cancer (CRC) and its high expression is associated with poor prognosis in CRC patients. We found that STC2 promoted resistance to anoikis in CRC by upregulating TGIF1 expression. Mechanistically, STC2 regulates TGIF1 expression by binding to its mRNA, thereby preventing its degradation. Our findings suggest that STC2 may serve as a potential therapeutic target for CRC treatment and offer new insights into the mechanisms underlying CRC development and progression. Future research should focus on the development of STC2-targeted therapies and understanding the broader impact of STC2 on other cancer types.

## Data Availability

The datasets presented in this study can be found in online repositories. The names of the repository/repositories and accession number(s) can be found in the article/Supplementary Material.
