# Supplementary material for: STC2 promotes anoikis resistance by modulating TGIF1 mRNA stability in colorectal cancer
Source: Front Cell Dev Biol. 2026 Jan 29;13:1695361. doi: 10.3389/fcell.2025.1695361 (PMC12894348; doi:10.3389/fcell.2025.1695361)

Cell Line Authentication Service

STR Profiling Report

Sample From: Shanghai HonSun Biological Technology Co., Ltd

Sample Type: Cell Line

Testing Method: STR Genotyping

Report Time: October 14, 2025

Cell Line Authentication – STR Profiling Report

Sample code

Table 1. Sample Code

Customer’s code	Company Code
HT-29	20251014-11

Sample Number:1

Sample Type: Cell line

Testing Type: STR

Testing Method:

DNA was extracted by a commercial kit from CORNING (AP-EMN-BL-GDNA-250G). The twenty STRs including Amelogenin locus were amplified by six multiplex PCR and separated on ABI 3730XL Genetic Analyzer. The signals were then analyzed by the software GeneMapper.

Data Interpretation:

Cell lines were authenticated using Short Tandem Repeat (STR) analysis asdescribed in 2012 in ANSI Standard (ASN-0002) by the ATCC Standards Development Organization (SDO) and in Capes-Davis et al., Match criteria for human cell line authentication: Where do we draw the line? Int J Cancer.2013;132(11):2510-9.

Test Results

1. STR profile

Table 2. STR and Amelogenin Genotyping Results of Cell line 20251014-11

Loci	Sample information			Cell Bank information		
	Sample name：HT-29			Cell line name：HT-29		
	**Allele1**	**Allele2**	**Allele3**	**Allele1**	**Allele2**	**Allele3**
D5S818	11	12		11	12	
D13S317	11	12		11	12	
D7S820	10	10		10	10	
D16S539	11	12		11	12	
VWA	17	19		17	19	
TH01	6	9		6	9	
AMEL	X	X		X	X	
TPOX	8	9		8	9	
CSF1PO	11	12		11	12	
FGA	20	22				
D21S11	29	30				
D18S51	13	13				
D8S1179	10	10				
D3S1358	15	17				
PENTAE	14	16				
PENTAD	11	13				

2. database annotation

Figure 1. STR matching analysis

EV	Cell No.	Cell name	Locus names								
			D5S818	D13S317	D7S820	D16S539	VWA	TH01	AM	TPOX	CSF1PO
		Query (Your Cell)	*11,12*	*11,12*	*10,10*	*11,12*	*17,19*	*6,9*	*x,x*	*8,9*	*11,12*
1.00(36/36)	299	HT-29	11,12	11,12	10,10	11,12	17,19	6,9	X,X	8,9	11,12

Note: The STR online match analysis of the test cell against DSMZ database, showing cell number (Cell No.) and cell name.

3. Authentication

☐ The submitted sample profile is human, but not a match for any profile in the DSMZ STR database.

☑ The submitted profile is an exact match for the following human cell line(s) in the DSMZ STR database (8 core loci plus Amelogenin): **HT-29** .

☐ The submitted profile is similar to the following DSMZ human cell line: /.

- **Note:** A cell line can be considered to be authenticated when 80% (exact match) of the alleles in its STR profile match profiles from tissue or other cell line samples from that donor or from database. Cell lines with between a 55% to 80% (similar) match require further profiling for investigation of relatedness.

Appendix:

1. Genotyping Strategy and Site Distribution

Table S1. Experimental Strategy and Sites

	Strategy 1	Strategy 2	Strategy 3	Strategy 4
1	AMEL	D16S539	TH01	D8S1179
2	D3S1358	D18S51	VWA	D12S391
3	D1S1656	D2S1338	D21S11	D19S433
4	D6S1043	CSF1PO	D7S820	FGA
5	D13S317	PENTAD	D5S818	
6	PENTAE		TPOX	

The allele match algorithm compares the 8 core loci plus amelogenin only, even though alleles from all lociwill be reported when available.

2. DSMZ tools was used to carry on the cell line comparison, which contains 2455 cell lines STR data from ATCC, DSMZ, JCRB ,ECACC， GNE and RIKEN databases. If the cell is not included in the above cell library, users need to compared with other databases.

Technician: Xiaolu Li

Checked by: Liqun Zhao

Issued by: Xue Han

Issue date: October 14, 2025

Applied Biosystems
GeneMapper Software 5

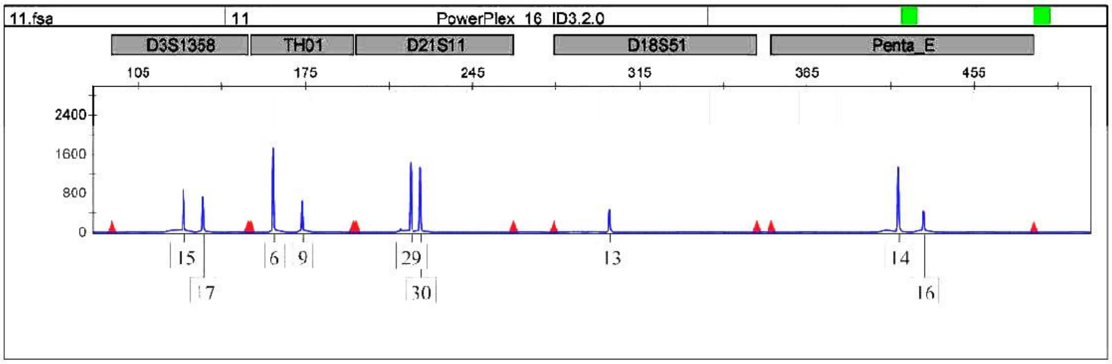

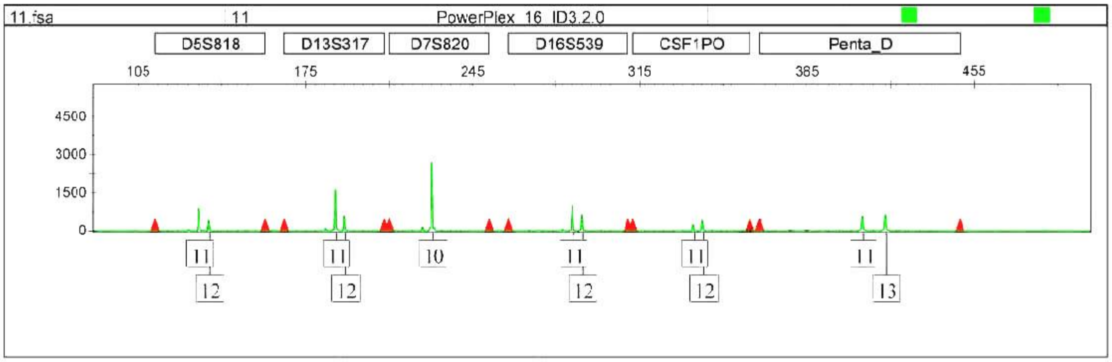

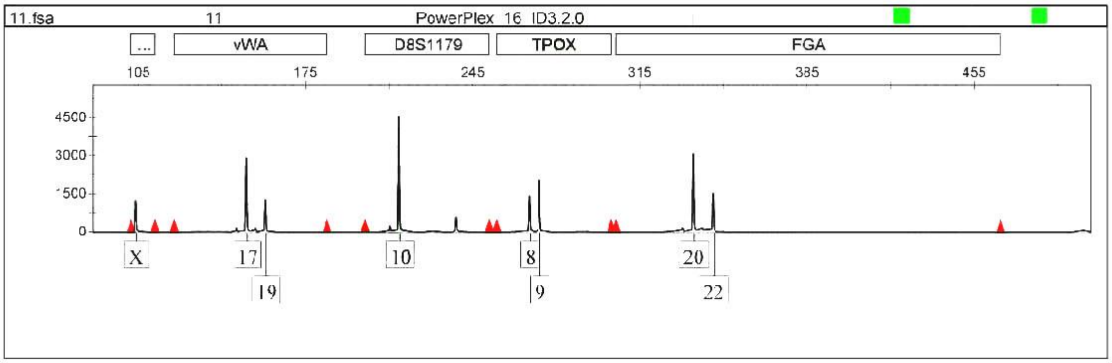

Supplement: Supplementary file 1 [file DataSheet2.pdf]
