# Supplementary figures and images for: STC2 promotes anoikis resistance by modulating TGIF1 mRNA stability in colorectal cancer
Source: Front Cell Dev Biol. 2026 Jan 29;13:1695361. doi: 10.3389/fcell.2025.1695361 (PMC12894348; doi:10.3389/fcell.2025.1695361)

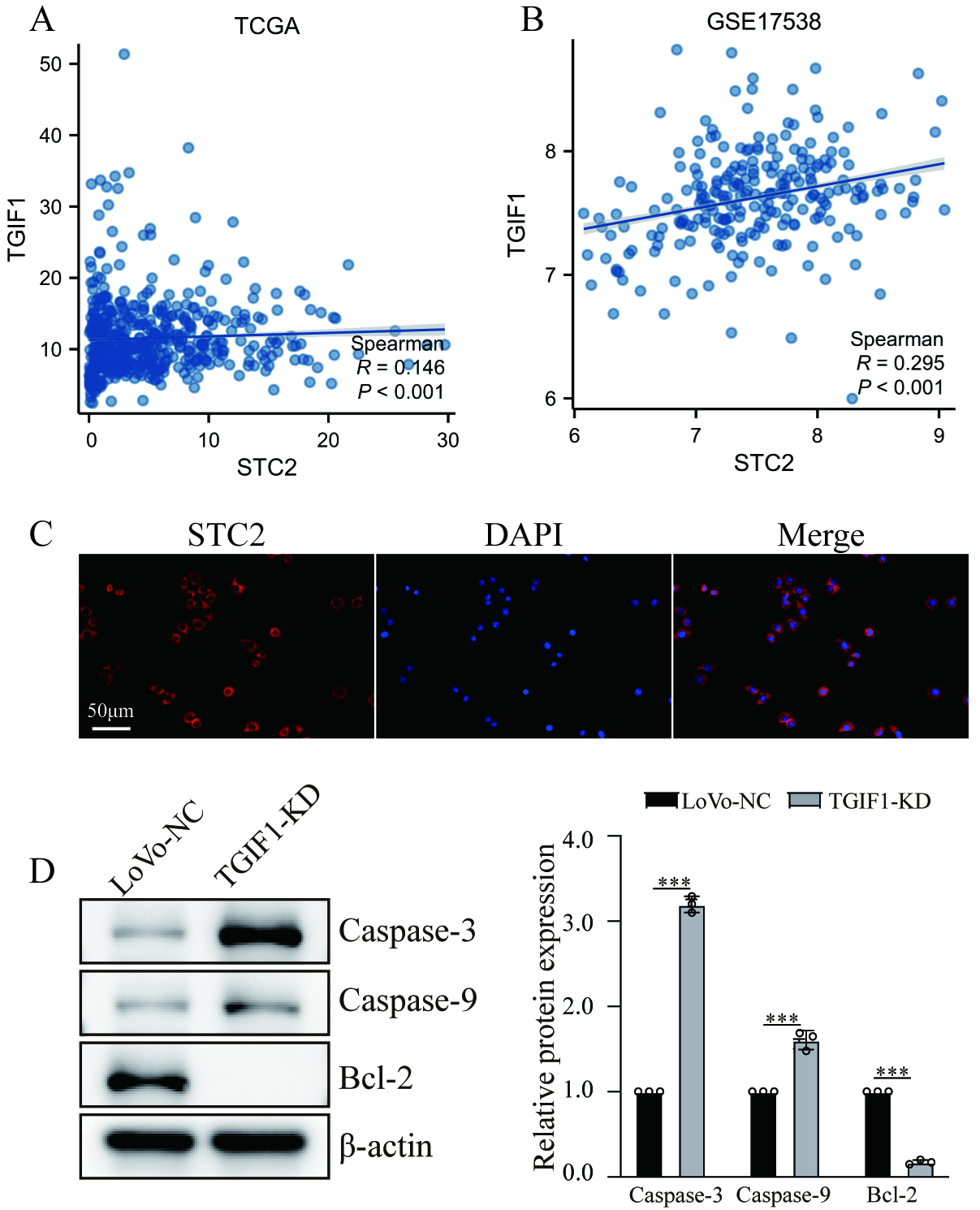

Supplement: Supplementary file 2 [file Image2.tif]

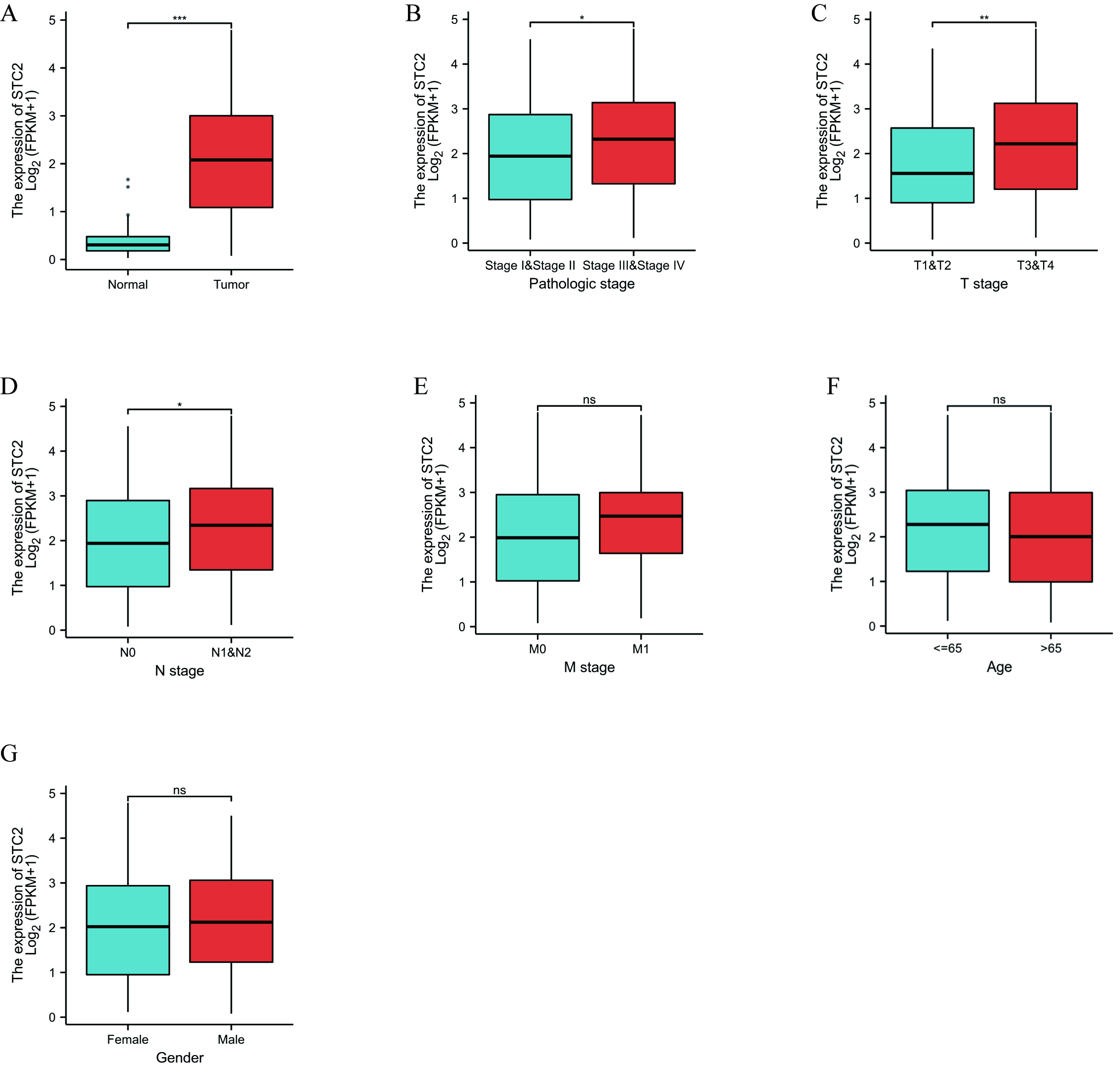

Supplement: Supplementary file 3 [file Image1.tif]
