# Supplementary material for: STC2 promotes anoikis resistance by modulating TGIF1 mRNA stability in colorectal cancer
Source: Front Cell Dev Biol. 2026 Jan 29;13:1695361. doi: 10.3389/fcell.2025.1695361 (PMC12894348; doi:10.3389/fcell.2025.1695361)
Supplement: Supplementary file 5 [file DataSheet1.docx]

**Supplementary Tables**

**Supplementary Table 1. Univariate and multivariate analyses of clinicopathologic parameters associated prognosis in TCGA**

| Characteristics | Total(N) | Univariate analysis | |  | Multivariate analysis | |
| --- | --- | --- | --- | --- | --- | --- |
|  |  | Hazard ratio (95% CI) | P value |  | Hazard ratio (95% CI) | P value |
| T stage | 476 |  |  |  |  |  |
| T3 | 322 | Reference |  |  |  |  |
| T4 | 60 | 2.717 (1.662-4.441) | **<0.001** |  | 2.339 (1.338-4.087) | **0.003** |
| T1 | 11 | 0.775 (0.190-3.160) | 0.722 |  | 4.043 (0.193-84.654) | 0.368 |
| T2 | 83 | 0.323 (0.130-0.802) | **0.015** |  | 0.740 (0.096-5.722) | 0.773 |
| N stage | 477 |  |  |  |  |  |
| N0 | 283 | Reference |  |  |  |  |
| N1 | 108 | 1.681 (1.019-2.771) | **0.042** |  | 0.228 (0.078-0.664) | **0.007** |
| N2 | 86 | 4.051 (2.593-6.329) | **<0.001** |  | 0.425 (0.152-1.186) | 0.102 |
| M stage | 414 |  |  |  |  |  |
| M0 | 348 | Reference |  |  |  |  |
| M1 | 66 | 4.193 (2.683-6.554) | **<0.001** |  | 47.670 (3.019-752.611) | **0.006** |
| Gender | 477 |  |  |  |  |  |
| Female | 226 | Reference |  |  |  |  |
| Male | 251 | 1.101 (0.746-1.625) | 0.627 |  |  |  |
| Age | 477 |  |  |  |  |  |
| <=65 | 194 | Reference |  |  |  |  |
| >65 | 283 | 1.610 (1.052-2.463) | **0.028** |  | 2.315 (1.400-3.830) | **0.001** |
| Pathologic stage | 466 |  |  |  |  |  |
| Stage I | 81 | Reference |  |  |  |  |
| Stage II | 186 | 2.035 (0.785-5.273) | 0.143 |  | 3.964 (0.288-54.484) | 0.303 |
| Stage III | 133 | 3.683 (1.436-9.448) | **0.007** |  | 26.646 (1.671-424.884) | **0.020** |
| Stage IV | 66 | 9.294 (3.608-23.936) | **<0.001** |  |  |  |
| STC2 | 477 | 1.207 (1.013-1.439) | **0.035** |  | 1.269 (1.039-1.550) | **0.019** |

**Supplementary Table 2.** **List of siRNAs**

| STC2 | siRNA.1: GAACAACUCUUGUGAGAUUTT  siRNA.2: UCAUCAAAGACGCCUUGAATT  siRNA.3: GCCAUCAGGGAAAUGGUGUTT |
| --- | --- |

**Supplementary Table 3. List of RT-PCR primers**

| STC2 | Forward: TGAAATGTAAGGCCCACGCT  Reverse: CGAGGTGCAGAAGCTCAAGA |
| --- | --- |
| TGIF1 | Forward: TGAGCACCGTTACAATGCCT  Reverse: GAAGTCCTGGTTGAGGTCCG |
| GAPDH | Forward: TTCACCACCATGGAGAAGGC  Reverse: GGCATGGACTGTGGTCATGA |

**Supplementary Table 4. List of antibodies**

| Antibody | Catalogue No. | company | Dilutions |
| --- | --- | --- | --- |
| STC2 | ab255610 | Abcam | 1:1000 |
| TGIF1 | ab52955 | Abcam | 1:1000 |
| Bcl-2 | 4223S | Cell Signaling Technology | 1:2000 |
| Caspase-3 | 14220S | Cell Signaling Technology | 1:1000 |
| Caspase-9 | 9502S | Cell Signaling Technology | 1:1000 |
| β-actin | ABL1010 | Abbkine | 1:5000 |
